# Synthesis of PNIPAAm-*g-*P4VP Microgel as Draw Agent in Forward Osmosis by RAFT Polymerization and Reverse Suspension Polymerization to Improve Water Flux

**DOI:** 10.3390/molecules27103214

**Published:** 2022-05-17

**Authors:** Yi Gao, Xuesong Yao, Qinggeng Jiang, Jianhe Liao, Yongping Chen, Rentong Yu

**Affiliations:** School of Materials Science and Engineering, Hainan University, Haikou 570228, China; yii_gao@163.com (Y.G.); 19085204210014@hainanu.edu.cn (X.Y.); qinggeng_j@163.com (Q.J.); 990359@hainanu.edu.cn (J.L.); chenyp@hainanu.edu.cn (Y.C.)

**Keywords:** microgel, forward osmosis, draw agent, P4VP

## Abstract

Microgels have unique and versatile properties allowing their use in forward osmosis areas as a draw agent. In this contribution, poly(4-vinylpyridine) (P4VP) was synthesized via RAFT polymerization and then grafted to a poly(*N*-Isopropylacrylamide) (PNIPAAm) crosslinking network by reverse suspension polymerization. P4VP was successfully obtained by the quasiliving polymerization with the result of nuclear magnetic resonance and gel permeation chromatography characterization. The particle size and particle size distribution of the PNIPAAm-*g-*P4VP microgels containing 0, 5, 10, 15 and 20 wt% P4VP were measured by means of a laser particle size analyzer. It was found that all the microgels were of micrometer scale and the particle size was increased with the P4VP load. Inter/intra-molecular-specific interactions, i.e., hydrogen bond interactions were then investigated by Fourier infrared spectroscopy. In addition, the water flux measurements showed that all the PNIPAAm-*g*-P4VP microgels can draw water more effectively than a blank PNIPAAm microgel. For the copolymer microgel incorporating 20 wt% P4VP, the water flux was measured to be 7.48 L∙m^−2^∙h^−1^.

## 1. Introduction

Water shortages have always posed a threat to living bodies around the world. By 2025, the proportion of the population lacking water will increase to two-thirds of the world's population [1,2]. In particular, the available water resources per capita are one quarter of the world’s average in China, and several cities have the problem of insufficient water supply, so it is of crucial importance to take action to alleviate the burden of water shortage. Fresh water is a very precious resource, and the reuse of freshwater paves the way for ensuring the water supply without the sacrifice of the natural freshwater ecosystem. With the rapid development of functional polymers since the 1980s, various water purification technologies such as nanofiltration, reverse osmosis, ion exchange membranes, photothermal evaporators, etc., have emerged and been widely applied [3]. Currently, most countries in the world mainly use reverse osmosis technology for seawater desalination [4]. The energy consumption of this technology is generally 3–4 KWh/m^3^. Although great progress has been achieved compared with phase-change seawater desalination technologies such as distillation, the problem of energy consumption is still troublesome [5,6,7].

Forward osmosis (FO) is a membrane separation process which utilizes the osmotic pressure gradient between the draw and the feed solution as the driving force without external pressure [8]. The FO process has the advantages of low energy consumption, non-contaminated performance of the membrane, and operation convenience, which has recently arose as a hotspot in the fields of drug release, renewable energy storage, desalination and concentrated brine treatment [9]. Draw agent and membrane technologies are key factors affecting the further application of FO technology, in which the ideal draw agent should have the characteristics of causing high osmotic pressure, preventing reverse diffusion, easy and rapid separation from water, reusability, non-toxicity, etc. [2,10,11,12,13,14,15,16]. Among the academic and engineering research findings of forward osmosis technology, inorganic-salt-type draw agents have been extensively developed [17]. New types of draw agents such as organic ion salt draw agents, polyelectrolyte draw agents, magnetic nanoparticles and polymer hydrogel extracting agents have been explored [18,19,20,21]. Yen SK et al. [22] found that 2-methylimidazole compounds as draw agents could produce an osmotic pressure of 35 MPa, and they obtained a high forward osmotic water flux of 2.5–6 L∙m^−2^∙h^−1^ (LMH). Moreover, the draw agents can be recovered by membrane distillation at 70 °C.

The preparation of a highly effective draw agent has always been an important orientation of FO research. In 2011, Dan Li et al. [23] synthesized a temperature-sensitive polymer hydrogel that could achieve a switch in water absorption and dehydration by temperature stimulation, although its maximum water flux was only 0.77 L∙m^−2^∙h^−1^ (LMH). Aijiao Zhou et al. [24] obtained a gel draw agent with dual response of temperature and magnetic field through mixing Fe_3_O_4_ into PNIPAAm-*co*-AMPS; however, the water flux only reached 0.26 LMH. The lower water fluxes mentioned above did not meet the requirements of the engineering application [25]. Hong et al. [26] synthesized a 2-Acrylamido-2-methyl-1-propane sulfonic acid/2-(Dimethylamino)ethyl methacrylate (AMPS/DMAEMA) gel that responded to the electric field, and the water flux could be as high as 2.09 LMH. As an advanced draw agent in the FO process, the strategy to increase water flux [27] and grab pure water [28] has become one of the areas of focus of forward osmosis research.

A micro-hydrogel, with three-dimensional crosslinking networks at the micrometer scale, is able to absorb water until the swelling equilibrium and expel water with the process of deswelling. In contrast, a nano-scale hydrogel could have a high uptake of water owing to its large specific surface area, which would be beneficial to achieve a high water flux. However, the recovery of nano-scale draw agents in the FO procedure would be ineffective in terms of the suspension state of the nano-scale particles. As for common hydrogels with a centimeter scale, the efficiency of dehydration would also be low with the consideration of thermodynamics. Therefore, it is essential to synthesize a micro-meter hydrogel to avoid the disadvantages mentioned above and achieve balanced FO properties for the purpose of engineering applications. On the other hand, it is valuable to trigger the recovery process of draw materials under a specific condition such as heat, electric, or magnetic fields. Of them, a thermal-responsive micro-hydrogel featured with the gel collapse temperature (GCT) [29] is a good choice in consideration of the convenience of processing. Thermo-sensitive micro-hydrogels can shrink or swell in response to the change in temperature, accompanied by a distinct volume change. For example, poly(*N*-isopropylacrylamide) (PNIPAAm) microgels [30] and poly (*N*-isopropylmethylacrylamide) microgels will shrink when the environmental temperature is above the GCT. The gel collapse temperature (GCT) of the PNIPAAm gel system is only 32 °C, which paves the way for the PNIPAAm draw agent to potentially be easily recovered with a small amount of thermal energy. Li et al. [31] concluded that thermo-responsive PNIPAAm hydrogels not only induced a higher water permeation rate in the FO process but also improved dehydration efficiency. Water flux is significantly related to the chemical potential of draw agents. Generally, hydrophilic materials will result in a large chemical potential difference between draw agents and water and then exhibit high water flux.

In this work, 4-vinylpyridine was quasiliving polymerized through the reversible addition-fragmentation chain transfer (RAFT) method to prepare the hydrophilic polymer chain-transfer agent (P4VP). Then, P4VP was reacted with the *N*-Isopropylacrylamide monomer to afford the PNIPAAm-*g*-P4VP microgel via reverse suspension polymerization. Nuclear magnetic resonance (^1^H-NMR), gel permeation chromatography (GPC), Fourier infrared spectroscopy (FT-IR), and laser particle size analyzer LPSA were adopted to characterize the synthesized products. Furthermore, the water flux was tested in order to measure its performance.

## 2. Results and Discussion

### 2.1. Synthesis and Characterization of the PNIPAAm-g-P4VP

**Scheme 1:**
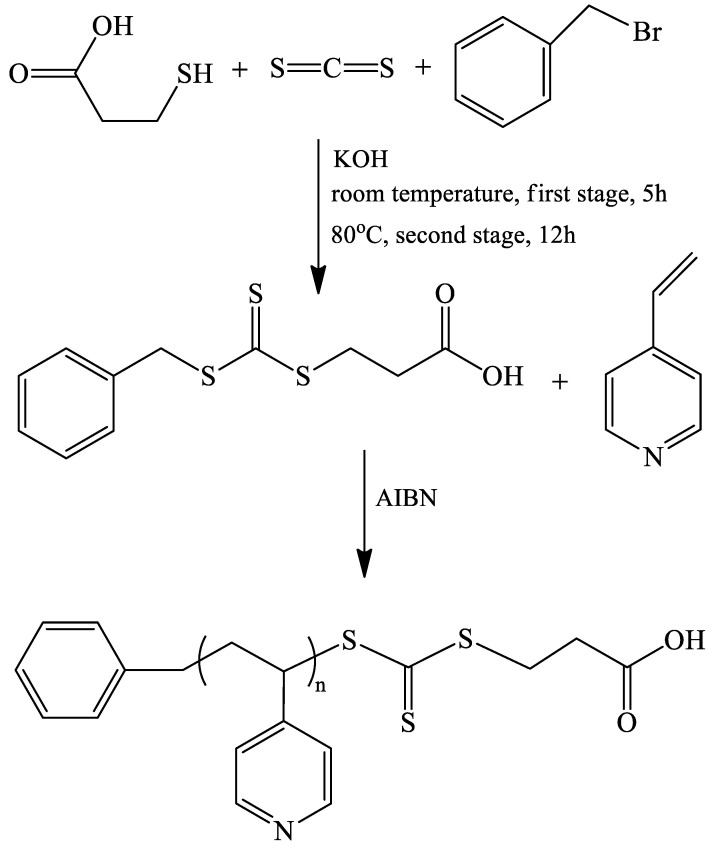

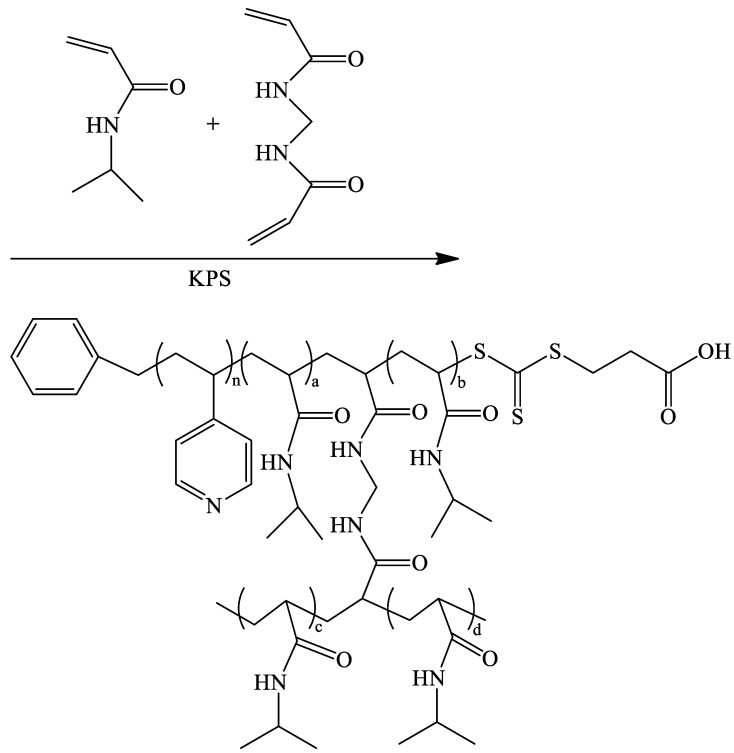
Synthesis routine of PNIPAAm-*g*-P4VP.

In this study, RAFT polymerization and reverse suspension polymerization were adopted to synthesize the PNIPAAm-*g*-P4VP microgel particles as the driving agent of forward osmosis. The synthesis process was presented, as shown in Scheme 1. At the beginning of this experiment, by adopting AIBN as the initiator and BSPA as the chain transfer agent, respectively, P4VP was synthesized via the reversible addition-fragmentation chain transfer (RAFT) polymerization of 4-vinylpyridine. By controlling the monomer conversion of 4-vinylpyridine, the P4VP with the targeted molecular weight was achieved.

**Figure 1:**
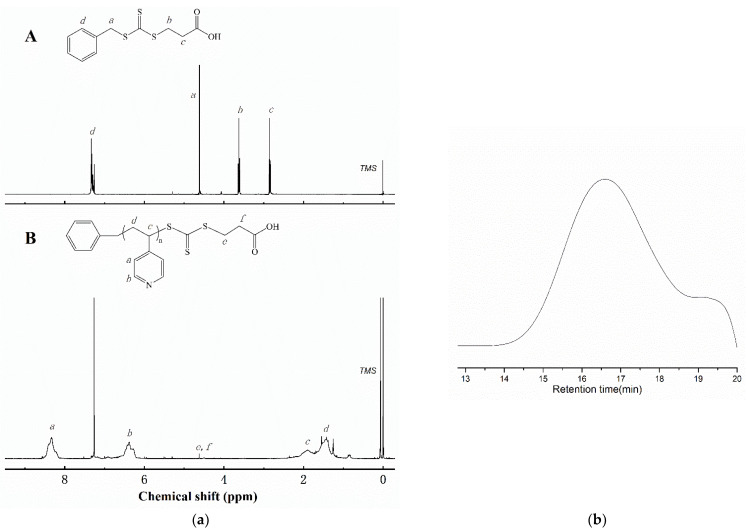
(**a**) ^1^H-NMR spectrum of the BSPA (A) and P4VP (B); (**b**) GPC traces of P4VP.

In Figure 1a, successfully synthesized BSPA (A) is reflected in the signal peaks at chemical shifts of 7.32 (multiplet), 4.61, 3.62, and 2.84 ppm, which are respectively attributed to the proton on the benzene ring and the methylene connected to the benzene ring, the trithiocarbonate group and the carboxyl group. As illustrated in Figure 1a, the resonance signals of P4VP (B) at 8.34 and 6.38 ppm are respectively attributed to the protons of the pyridine ring, and the resonance signals of P4VP (B) at 4.62, 1.92 and 1.89 ppm are respectively attributed to the protons of methylene, methine and methylene of the polycyclic aromatic hydrocarbons, which are respectively connected to trithiocarbonate and the pyridine ring and carboxyl group. It is estimated that the *(*$\bar{M_{n}}$) of P4VP -CTA is about 2200 Da. The monomer conversion of 4VP is 31%. This is close to the value calculated from the monomer conversion, indicating that the polymerization was a quasiliving polymerization [32,33]. Figure 1b shows the GPC curve of the P4VP long-chain molecule. GPC shows that the number–average molecular weight ($\bar{M_{n}}$) is 2210, and its polydispersity index is 1.47. In order to ensure the same contangle of the pendent chains grafted to the crosslink network, P4VP was synthesized by RAFT polymerization, i.e., quasiliving polymerization. Nevertheless, the molecular weight distribution is not perfect. In fact, the molecular weight distribution of polyelectrolytes synthesized via RAFT polymerization tends to be broad. For example, the polydispersity index of poly[[*N*-isopropylacrylamide]-*b*-acrylic acid-*b*-[*N*-isopropylacrylamide]] synthesized by Kamperman et al. was 1.59 [34]. The interaction equilibrium derived from inter- and inter molecular hydrogen bonds could be achieved competitively when P4VP was fully dissolved in DMF. However, when the DMF/P4VP solution was characterized with GPC, the interaction equilibrium could be disproportionated locally and thus have an effect on the hydrodynamic radius of the polymer.

With the grafting reaction of P4VP, PNIPAAm-*g*-P4VP microgel particles were synthesized by reverse suspension polymerization using BIS as the cross-linking agent and KPS as the initiator. It was observed that as the polymerization reaction proceeded, the viscosity of the system gradually increased, demonstrating that the degree of polymerization increased accordingly. As shown in Table 1, the feed ratios for each PNIPAAm-*g*-P4VP copolymer microgel network were summarized.

**Figure 2:**
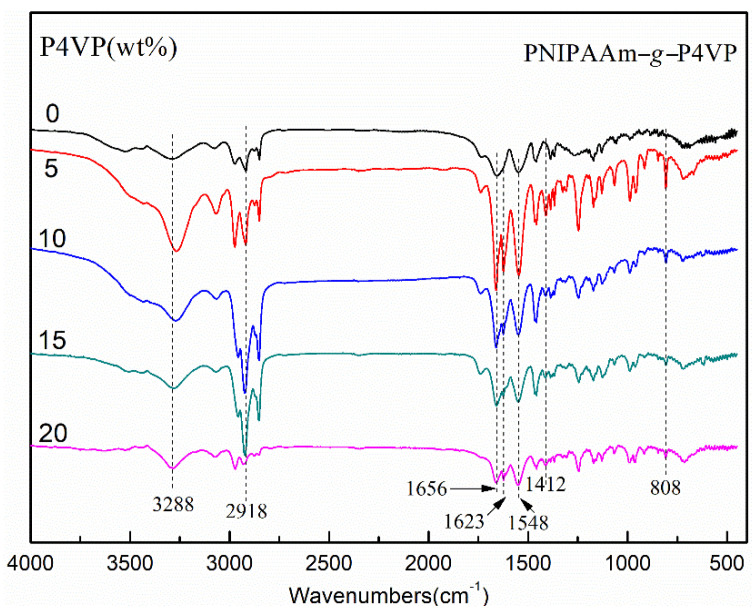
FTIR spectra of PNIPAAm and PNIPAAm−*g−*P4VP.

Figure 2 presents the FTIR spectra of a pure PNIPAAm microgel and PNIPAAm-*g*-P4VP copolymer microgels. It can be seen from the infrared spectra that the band at 1056 cm^−1^ corresponds to the characteristic of the stretching vibration of the carbon–sulfur double bond, as well as the vibration absorption band of the C-S bond appearing at 1243 cm^−1^. Combined with the NMR spectra, it can be concluded that the chain transfer agent we designed was successfully obtained. In addition, it indicates that the carbon-sulfur double bonds have not been destroyed during the polymerization reaction because the characteristic band of C-S double bonds all appear at approximately 1056 cm^−1^ in the figures. There are characteristic absorption bands of the pyridine ring at 1412 cm^−1^ and 1623 cm^−1^, and a sharp band at 808 cm^−1^ corresponds to the absorption band of the mono-substituted pyridine ring. The structure of the amide is explicitly verified by the N–H stretching vibration peaks at 3288 cm^−1^ and -CH_2_ stretching vibration peaks at 2918 cm^−1^. Otherwise, an amide I band at 1656 cm^−1^ is assigned to the C=O stretching vibration absorption peak, as well as an amide II band at 1548 cm^−1^ being an N–H bending vibration peak.

The results above prove that P4VP was successfully grafted onto the PNIPAAm network. Meanwhile, the lower frequency stretching vibration band (1623 cm^−1^) appears near 1656 cm^−1^. This spectral feature shows that intermolecular interactions between PNIPAAm and P4VP were formed through hydrogen bond interactions. In addition, by comparing the band intensity between 1656 cm^−1^ and 1623 cm^−1^, it is found that the hydrogen bond interactions between PNIPAAm and P4VP are enhanced with the increase in P4VP.

### 2.2. Analysis of Particle Size

**Figure 3:**
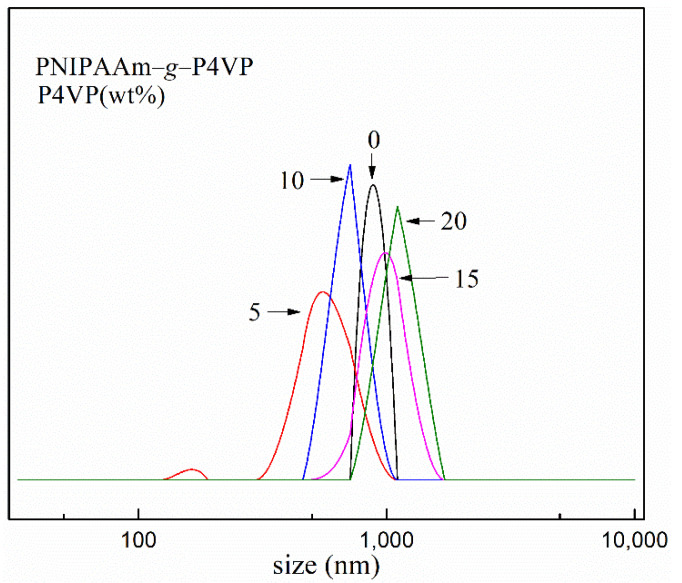
Particle size distribution of PNIPAAm microgel modified by P4VP.

The size averages and particle size distribution of the PNIPAAm based microgels modified with different loads of 4-vinylpyridine are presented in Figure 3. It can be observed that all of the particle sizes are of micrometer scale and the particle size increases with the load of P4VP. For the microgel incorporating 5 wt% P4VP, the particle size is 548 nm. The particle size of PNIPAAm-*g*-P4VP containing 10 wt% P4VP is increased to 713 nm. When the P4VP load increases further, the particle size would be 996 nm and 1118 nm for microgels containing 15 wt% and 20 wt% P4VP, respectively. At the same time, the particle size of blank PNIPAAm gel is 886 nm.

P4VP is insoluble in water until the pH is less than 4.5. With the protonation of pyridine groups, a transition from hydrophobic to hydrophilic states will take place quickly [35]. P4VP moieties of the microgel will not be hydrated and will thus have a negative effect on the swelling ratio of the microgel. As a result, the particle size of a microgel containing 5 wt% or 10 wt% P4VP should be smaller than that of a blank PNIPAAm microgel. However, as mentioned above, the intensified hydrogen bond interactions between PNIPAAm and 15 wt% or 20 wt% P4VP provide a different condition, i.e., P4VP moieties can be protonated by accepting more H^+^ from PNIPAAm and extended more effectively in the water environment. Accordingly, the particle size will be increased in consideration of swelling.

### 2.3. Test and Analysis of Water Flux

**Figure 4:**
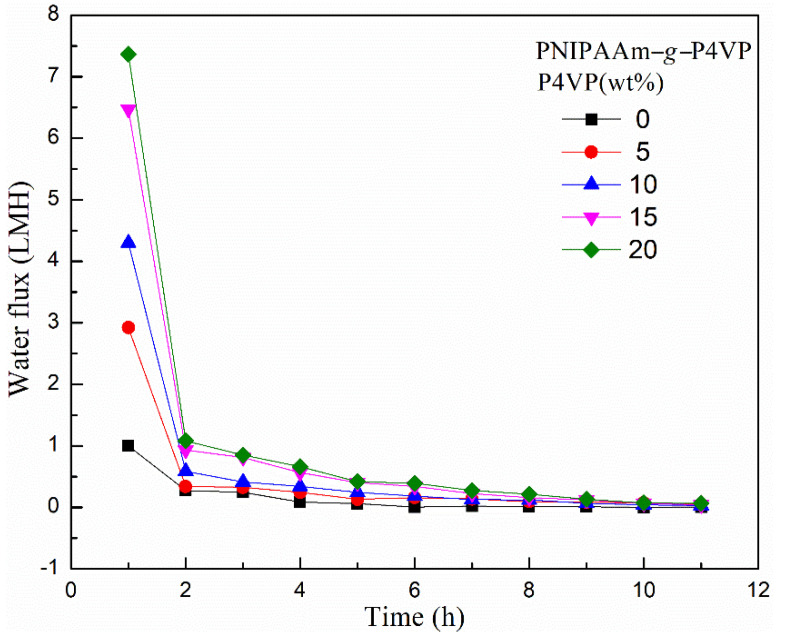
Water flux measurement graph of PNIPAAm microgel modified with P4VP.

The effect of PNIPAAm microgels containing different proportions of P4VP on water flux was investigated, and the results are shown in Figure 4. For all of the samples, the maximum water flux can be observed in the first hour. When the treatment times last longer, the values of the water flux tended downward and the plummets would always be found at the processing time of 2 h. Moreover, the value of water flux can be improved significantly with an increase in P4VP content, with the characteristics of rapid water permeation. For the microgel draw materials incorporating 20 wt% P4VP, the water flux was measured to be 7.48 LMH, far higher than that of blank PNIPAAm microgel draw agent. The microgels featured a cross-linking network structure and would swell rather than dissolve in water like other types of draw agents. Hu et al. clarified that water flux was essentially driven by the water chemical potential or water activities. Strong interactions between hydrogels composed of polyelectrolytes and water could reduce the water potential and then result in high water flux [18]. In this work, the hydration and ionization of P4VP was believed to be the cause of the marked difference in water flux. In addition, multivalent ionic solutes have been considered as favorable draw agents on account of more ionic species. P4VP was grafted to the three-dimensional PNIPAAm network in our work. Therefore, there were lots of anionic-type polyelectrolytes tethered with the network, which would accelerate water absorption and improve the performance of water flux. Furthermore, compared with common hydrogels with a latitude of centimeters, microgels with sizes between 700 nm and 1118 nm could grab water effectively due to their large specific surface area.

## 3. Materials and Methods

### 3.1. Materials

2, 2-Azobisisbutyronitrile (AIBN; J&K Scientific, Beijing, China) was purified by recrystallizing ethanol twice. 4-vinylpyridine (95%; Amethyst, Beijing, China) was refined by removing the inhibitor with an alkaline alumina column before use. The alkaline alumina was purchased from the Sinopharm Chemical Reagent Company. *N*-Isopropylacrylamide (NIPAAm; ≥98%; Accela, Beijing, China) was purified by recrystallization from petroleum ether. *N*,*N*’-methylenebisacrylamide (BIS; ≥99%; J&K Scientific, Beijing, China), 3-Mercaptopropionic acid (99%; Aladdin-Reagent, Shanghai, China) and benzyl bromide (≥98%; Amethyst, Beijing, China) were used without further purification. Carbon disulfide (≥99.9%) was pure analytical grade and was provided by the Sinopharm Reagent Company. Pyridine and benzyl bromide were of a chemically pure grade. All the other reagents were of analytical grade and used as received. Methanol was purified via distillation.

### 3.2. Synthesis of 3-Benzylsulfanylthiocarbonylsufanylpropionic Acid (BSPA)

The procedure for synthesizing 3-Benzylsulfanylthiocarbonylsufanylpropionic acid (BSPA) was followed from Stenzel’s contribution [36]. In the first step, 3-mercaptopropionic acid (20 mL, 0.23 mol) was added into the aqueous solution of potassium hydroxide (KOH) (25.8 g, 0.46 mol), and then 30 mL carbon disulfide (CS_2_) was slowly introduced under magnetic stirring within 30 min. In this state, after magnetic stirring for a further 5 h, benzyl bromide (39.6 g, 0.23 mol) was added, heated up to 80 °C, and the reaction system was kept under vigorous stirring for 12 h. Afterwards, the system was chilled to 25 °C and 300 mL of chloroform was introduced for the purpose of dissolving the intermediate. An acidifying reaction was then carried out by dropping an aqueous solution of hydrochloric acid, and the operation was continued until the organic layer turned bright yellow. The role of chloroform (2 × 100 mL) here was to isolate the product from the aqueous phase. The combined organic phase was further washed with 10 wt% sodium carbonate (Na_2_CO_3_) (3 × 100 mL), 100 mL of Na_2_CO_3_ each time, and then extracted with chloroform, followed by drying overnight with anhydrous magnesium sulfate (MgSO_4_). After filtration and recrystallization in dichloromethane, the yellow crystals were obtained with a yield of 90%.

### 3.3. Synthesis of Poly(4-vinylpyridine) (P4VP)

The preparation of poly(4-vinylpyridine), viz. P4VP was performed via RAFT polymerization, in which BSPA was used as the chain transfer agent. 4-VP (6.2445 g, 59.39 mmol), BSPA (0.6093 g, 2.24 mmol), AIBN (0.0734 g, 0.447 mmol), 2.4 mL of methanol and 0.6 mL of deionized water were added into a 50 mL anhydrous round-bottomed flask and agitated by magnetic stirring. The system was equipped with a standard Schlenk line to remove oxygen (four cycles of freeze–thaw–exhaust–thaw) and then reacted under the protection of nitrogen at 60 °C for 22 h. Methanol and ethyl ether were then used to dissolve and precipitate the product, respectively. This purification procedure was repeated thrice, and the final precipitate was dried in a 30 °C vacuum oven for 24 h to obtain the brown-red P4VP powder.

### 3.4. Preparation of PNIPAAm-g-P4VP Polymer Micro-Hydrogel

The P4VP was employed to synthesize the PNIPAAm-*g*-P4VP microgel through the method of reverse suspension polymerization. Typically, different weights of P4VP -CTA monomers were taken as the control. NIPAAm (1.58 g, 14 mmol), BIS (0.0214g, 0.14 mmol), and P4VP (0 g, 0.1 g, 0.2 g, 0.3 g, 0.4 g) were used as the dispersed phase in aqueous solutions. An organic solution composed of cyclohexane and Span 60 (20%) was used as the continuous phase. The aqueous solution of potassium persulfate (KPS) (0.0029 g, 0.0082 g, 0.0123 g, 0.0164 g) was prepared according to the different monomer weight to initiate the crosslinking reaction, respectively. The continuous phase and the dispersed phase were both bubbled with nitrogen for 30 min before mixing. The continuous phase was then heated to 45 °C under nitrogen protection. After the continuous phase was mixed with the dispersed phase, the pre-prepared KPS aqueous solution was injected into the reaction system and heated to 75 °C and reacted for 24 h. The products obtained from the reaction were rotary evaporated to remove the organic solvent, and then the final product PNIPAAm-*g*-P4VP micro-hydrogels were achieved by a lyophilizer. The resultant products were light yellow, and the yellow deepened with the increase in P4VP. The products were stored in a vacuum oven at 30 °C for 24 h.

### 3.5. Measurement and Characterization

^1^H-NMR spectra were acquired on a Varian Mercury Plus 400 MHz nuclear magnetic resonance spectrometer. P4VP was dissolved in deuterated chloroform (CDCl_3_) with tetramethylsilane (TMS), which was used as the internal reference. The infrared spectra were recorded on a Thermo Scientific Nicolet IS10 Fourier transform infrared spectrometer with a resolution of 2 cm^−1^, and all the spectra were recorded in the wavenumber range of 4000–500 cm^−1^ by accumulating 64 scans. The synthesized micro-hydrogel samples were evenly blended with anhydrous KBr power and pressed to prepare the small flakes until all the flakes were thin enough to comply with the Beer-Lambert law. The number-average molecular weight and polydispersity index were investigated by gel permeation chromatography (GPC) on Waters 1515 GPC, in which the standard curve was calibrated with standard polyethylene glycol. The sample was dissolved in *N*,*N*-Dimethyl formamide (DMF) with a concentration of 3 mg/mL, and the liquid flow speed was controlled at 2.5 µL/min to pass through the double column. The particle size of the micro-hydrogels was characterized by a zetasizer Nano S90 Laser Particle Size Analyzer of Malvern co., ltd (LPSA), reflecting the particle size as well as particle size distribution. Before the test, the obtained sample powder needed to be swollen in deionized water, and each test was continued for three minutes for balance consideration.

**Figure 5:**
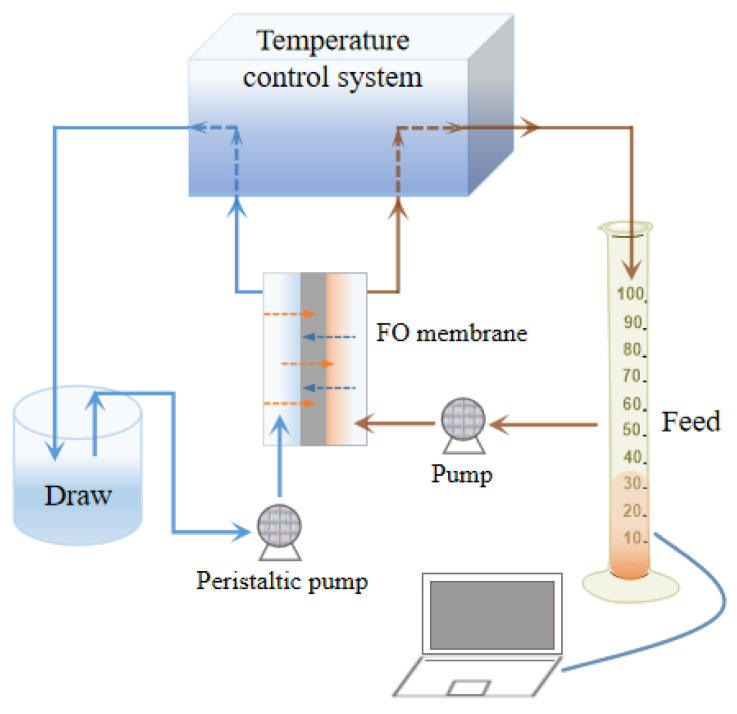
Water flux measurement graph of PNIPAAm microgel modified with P4VP.

The water flux was measured with a device that was mainly composed of feed solution, draw solution, and HTI cellulose triacetate (CTA) osmotic membrane, as depicted in Figure 5. Prior to testing, the membrane was immersed in a 2000 ppm NaCl aqueous solution for 24 h. At room temperature (25 °C), a peristaltic pump was used to form a circulating flow of the raw material liquid in the channels of the permeable membrane, and the flow of the liquid on both sides was adjusted by a flow meter to control the flow rate to 30 mL/min. The raw material liquid cylinder was placed on an electronic balance to calculate the water flux. The obtained powdered microgel was flatly spread on the front of the CTA membrane. A peristaltic pump was in gear to pass the NaCl aqueous solution into the membrane after fixing the membrane module. Once the first drop of the aqueous solution returned to the raw material tank, the first number was read, and the timing was started simultaneously. The weight of the water was recorded every 1 h within 12 h. The water flux ($J_{w}$, L∙m^−2^∙h^−1^, LMH) was calculated by using Equation (1):

This is example 1 of an equation:

(1) $$J_{w}=\frac{\Delta V}{A\Delta t}$$

where ∆*V* is the water volume that passed through the permeable membrane (L); *A* denotes the effective membrane area (m^2^) of the membrane; and ∆*t* is the unit time (h).

## 4. Conclusions

4-vinylpyridine was radically addition-fragmentation polymerized, and the afforded poly(4-vinylpyridine) (P4VP) was characterized by means of nuclear magnetic resonance and gel permeation chromatography. The results demonstrated that the P4VP was achieved via quasiliving polymerization. Reverse suspension polymerization was then carried out to prepare the PNIPAAm microgel grafted with pendent P4VP chains. The particle size of the PNIPAAm-*g-*P4VP microgels was found to be increased with the increase in P4VP content. At the same time, all of the particle sizes were of the micrometer scale. The difference in particle size can be interpreted with consideration of the inter/intra-molecular specific interactions. In essence, P4VP moieties could be extended more effectively in water environments. In addition, water flux was measured to be increased significantly by using the PNIPAAm-*g*-P4VP microgels as a draw agent. The water flux reached 7.48 LMH for the PNIPAAm-*g*-P4VP draw system containing 20 wt% P4VP. This work would be inspiring for the preparation of novel draw agents on account of the characteristics of P4VP. P4VP is not only a pH-responsive polyelectrolyte, but also an antibacterial polymer. Further research will be explored in the near future.

## Figures and Tables

**Table 1:** The feed ratios of the crosslinking reaction for preparation of PNIPAAm-*g*-P4VP.

Sample Name	P4VP in Feed	NIPAAm	P4VP	NIPAAm: P4VP
	(wt%)	(g)	(g)	(Molar Ratio)
PNIPAAm	0	1.58(≈1.60)	0	-
PNIPAAm-*g*-P4VP 5	5		0.1	16:1
PNIPAAm-*g*-P4VP 10	10		0.2	8:1 (16:2)
PNIPAAm-*g*-P4VP 15	15		0.3	16:3
PNIPAAm-*g*-P4VP 20	20		0.4	4:1 (16:4)

## Data Availability

Not applicable.

## Sample Availability

Samples of the compounds are available from the authors.
